# Relative fat mass, a new index of adiposity, is strongly associated with incident heart failure: data from PREVEND

**DOI:** 10.1038/s41598-021-02409-6

**Published:** 2022-01-07

**Authors:** Navin Suthahar, Laura M. G. Meems, Coenraad Withaar, Thomas M. Gorter, Lyanne M. Kieneker, Ron T. Gansevoort, Stephan J. L. Bakker, Dirk J. van Veldhuisen, Rudolf A. de Boer

**Affiliations:** 1 Department of Cardiology, University Medical Center Groningen, University of Groningen, Groningen, The Netherlands; 2 Division of Nephrology, Department of Internal Medicine, University Medical Center Groningen, University of Groningen, Groningen, The Netherlands

**Keywords:** Cardiology, Risk factors

## Abstract

Body-mass index (BMI), waist circumference, and waist-hip ratio are commonly used anthropometric indices of adiposity. However, over the past 10 years, several new anthropometric indices were developed, that more accurately correlated with body fat distribution and total fat mass. They include relative fat mass (RFM), body-roundness index (BRI), weight-adjusted-waist index and body-shape index (BSI). In the current study, we included 8295 adults from the PREVEND (Prevention of Renal and Vascular End-Stage Disease) observational cohort (the Netherlands), and sought to examine associations of novel as well as established adiposity indices with incident heart failure (HF). The mean age of study population was 50 ± 13 years, and approximately 50% (n = 4134) were women. Over a 11 year period, 363 HF events occurred, resulting in an overall incidence rate of 3.88 per 1000 person-years. We found that all indices of adiposity (except BSI) were significantly associated with incident HF in the total population (P < 0.001); these associations were not modified by sex (P interaction > 0.1). Amongst adiposity indices, the strongest association was observed with RFM [hazard ratio (HR) 1.67 per 1 SD increase; 95% confidence interval (CI) 1.37–2.04]. This trend persisted across multiple age groups and BMI categories, and across HF subtypes [HR: 1.76, 95% CI 1.26–2.45 for HF with preserved ejection fraction; HR 1.61, 95% CI 1.25–2.06 for HF with reduced ejection fraction]. We also found that all adiposity indices (except BSI) improved the fit of a clinical HF model; improvements were, however, most evident after adding RFM and BRI (reduction in Akaike information criteria: 24.4 and 26.5 respectively). In conclusion, we report that amongst multiple anthropometric indicators of adiposity, RFM displayed the strongest association with HF risk in Dutch community dwellers. Future studies should examine the value of including RFM in HF risk prediction models.

## Introduction

The worldwide prevalence of obesity has nearly tripled during the last 50 years^1^, and the burden of obesity is expected to increase even further in the coming decade^2,3^. Heart failure (HF) is also an emerging epidemic, with lifetime risk estimates between 20 and 33% in both sexes^4^. Excessive adipose tissue accumulation increases the risk of developing HF^5–7^, and recent data indicate that among modifiable risk factors, obesity explained a substantial proportion of incident HF in the general population^8,9^.

The current definition of overweight and obesity is based on body-mass index (BMI), even though it is known that BMI may not accurately reflect fat mass^10^. Interestingly, anthropometric measures such as waist circumference (WC) and waist-to-hip ratio (WHR), that more strongly relate with abdominal fat distribution, were not found to be substantially better than BMI in predicting HF risk^11,12^. With obesity becoming more prevalent and likely becoming a major driver of HF risk, there is a growing need for more “adequate but easy-to-measure” surrogates of fat mass that also better associate with future cardiovascular risk.

Over the past 10 years, several indices of adiposity were developed, that more accurately correlated with body fat distribution and total fat mass. They include body shape index (BSI)^13^, body roundness index (BRI)^14^, weight-adjusted-weight index (WWI)^15^ and relative fat mass (RFM)^16^. In the current study, we sought to examine for the first time the association between newly developed indices of adiposity and the risk of incident HF in community-dwelling individuals.

## Methods

**Figure 1:**
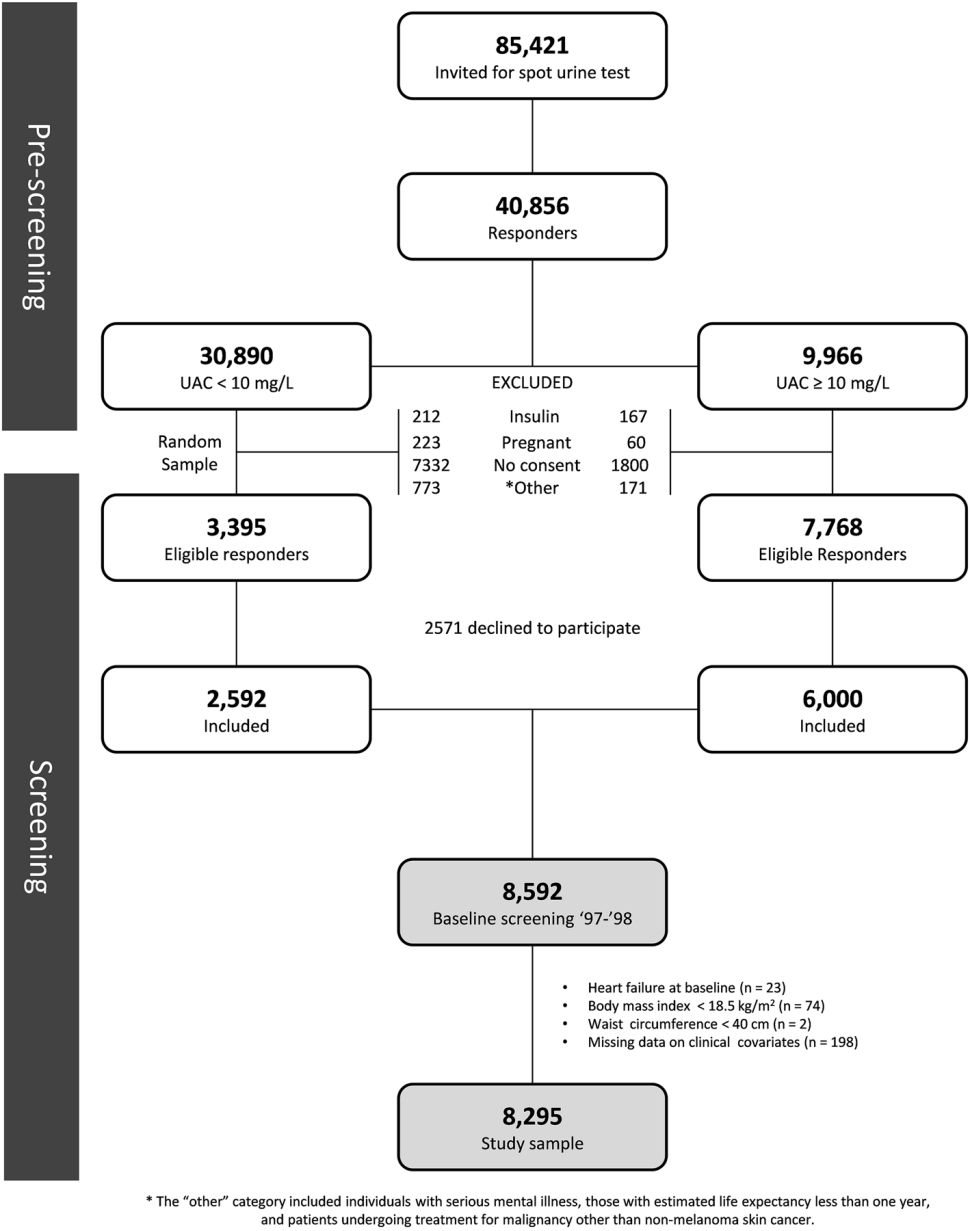
Flow diagram: participant selection in the PREVEND study.

The PREVEND (Prevention of Renal and Vascular End-Stage Disease) study (1997–1998) is a Dutch cohort taken from the general population of Groningen, the Netherlands^17–19^. This study was designed to prospectively evaluate whether increased urinary albumin excretion (UAE > 10 mg/L) in community-dwelling individuals was associated with cardiovascular and renal disease^20^. In brief, all inhabitants from Groningen, aged 28 to 75 years were asked to respond to a short questionnaire and provide early-morning urine samples (N = 85,421). The response rate was 48% (n = 40,856). Responders with UAE ≥ 10 mg/L (n = 7786) as well as a randomly selected control group with UAE < 10 mg/L (n = 3395) were invited to the outpatient clinic for comprehensive health assessment including responding to questionnaires, anthropometric measurements, fasting blood draw, and urine sampling^19^. Individuals with type-1 diabetes (defined as insulin requirement), pregnant women (self-reported), and unwilling subjects were excluded from the study^19^. A final total of 6000 individuals with UAE ≥ 10 mg/L and 2592 individuals with UAE < 10 mg/L constitute the baseline PREVEND cohort (N = 8592)^19^. From the baseline cohort, we excluded participants with HF at baseline according to hospital records (n = 23), BMI < 18.5 kg/m^2^ (n = 74), WC < 40 cm (n = 2) and missing covariates (n = 198), resulting in a total of 8295 participants available for analysis (Fig. 1). The current study conformed to the principles drafted in the Helsinki declaration and was approved by the medical ethical committee of the University Medical Center Groningen (UMCG). Written informed consent was received from all participants.

### Baseline measurements

Body weight, height, WC and hip circumference (HC) were measured in a standing position during the baseline visit. WC was measured midway between the lowest rib and the iliac crest at the end of expiration. HC was measured at the widest portion at the level of greater trochanters. BMI was calculated as the ratio between weight and height-squared^11,12^; overweight was defined as BMI between 25 and 30 kg/m^2^ and obesity as BMI ≥ 30 kg/m^2^; WHR was the ratio between WC and HC^11,12^; BSI was calculated as: [1000 × WC × Weight^-2/3^ × Height^5/6^]^13^; BRI was calculated as: [364.2–365.5 × (1 − ((0.5 × WC/π)^2^/(0.5 × Height)^2^))^0.5^]^14^; WWI was calculated as: [(WC × 100)/(Weight^0.5^)]^15^; and RFM was calculated as: [64 − (20 × Height/WC) + (12 × sex), with sex = 0 (men), and sex = 1 (women)]^16^.

Smoking was defined as self-reported current smoking or smoking cessation within the previous year. Hypertension was defined as systolic BP (SBP) ≥ 140 mm Hg, diastolic BP (DBP) ≥ 90 mm Hg or self-reported antihypertensive medication usage. Blood pressure (BP) was measured ten times during 10 min using an automatic Dinamap XL Model 9300 series; BP was calculated as the mean of the last two measurements^19^. Type-2 diabetes was defined as a fasting plasma glucose ≥ 7.0 mmol/L (126 mg/dL), random plasma glucose ≥ 11.1 mmol/L (200 mg/dL), self-reporting of a physician diagnosis or record of glucose-lowering medication use obtained from central pharmacy registry^19^. History of myocardial infarction and cerebrovascular accident were based on individuals’ medical history derived from a structured questionnaire i.e., hospitalization ≥ 3 days as a result of this condition; this was complemented by a review of the medical report^19^. Individuals with AF at baseline screening were considered to have prevalent AF. Total cholesterol and plasma glucose were measured by a dry chemistry method (Eastman Kodak, Rochester, New York).

### Incident heart failure

Individuals were prospectively followed for the first occurrence of HF or death within 13.5 years of baseline examination. HF records including dates were retrieved from clinical charts. Individuals suspected of having HF were identified according to European Society of Cardiology (ESC) guidelines^21^. An endpoint adjudication committee of seven independent HF experts further evaluated these selected individuals, and two different experts validated each case. A joint decision was made within the committee in the case of disagreement. Based on left ventricular ejection fraction (LVEF) cutpoint of 50%^22^, HF was subcategorized into HF with reduced EF (HFrEF) or preserved EF (HFpEF); LVEF was available for all HF cases. Further details can be found elsewhere^17^.

### Statistical analyses

Continuous data are presented as medians, Q1-Q3 (50th percentile, 25th–75th percentile) and categorical variables are represented as percentages. For further analyses, all continuous adiposity measures were standardized. In primary analyses, we examined associations of adiposity indices with incident HF in the total population using Cox regression models adjusting initially for age and sex, and subsequently also for smoking, glucose, cholesterol, systolic blood pressure^23^, history of myocardial infarction, stroke and atrial fibrillation. We tested for *adiposity index × sex* terms in the multivariable model. A multiple testing corrected P-value of 0.007 (0.05/7, Bonferroni adjustment) and an interaction P-value (*P*_*int*_) of 0.1 denoted statistical significance. We also examined the shape of associations of adiposity indices with incident HF using multivariable fractional polynomial models. In secondary analyses, we examined associations of adiposity indices with incident HF according to pre-specified age categories (young < 55 years; middle aged 55–65 years; and old ≥ 65 years), and across BMI categories (lean < 25 kg/m^2^; overweight 25–30 kg/m^2^; and obese ≥ 30 kg/m^2^). Additionally, we evaluated associations of adiposity indices with HFrEF and HFpEF separately. Finally, we used Harrell’s C-statistic, Akaike information criteria (AIC)^24,25^ and P-values based on likelihood ratio (LHR) test^22^ to examine the incremental predictive value of adiposity indices (beyond a clinical model) for HF and its subtypes. A P_LHR_ < 0.05 was considered as moderate evidence against the null hypothesis, and a P_LHR_ < 0.01 was considered as strong evidence against the null hypothesis^26^. All statistical analyses were performed using STATA version-14.

## Results

Mean age of the cohort was 49.8 ± 12.6 years and 49.8% (n = 4134) were women. Around 41% of the population was overweight (n = 3416) of which 42% were women, and 16% was obese (n = 1311) of which 54% were women (Table 1). Sex-specific distributions of adiposity indices are shown in Supplementary Table S1. Most adiposity indices showed moderate to strong correlations with each other, and with age in both sexes (Supplementary Table S2).

**Table 1:** Baseline characteristics of PREVEND participants.

	Total population (n = 8295)	Men (n = 4161)	Women (n = 4134)
**Clinical characteristics**			
Age, years	48.5 (39.2, 60.2)	49.8 (40.2, 62.3)	47.4 (38.4, 57.9)
Smoking	3124 (37.8%)	1573 (37.9%)	1551 (37.6%)
Overweight (BMI 25–30 kg/m^2^)	3416 (41.2%)	1991 (47.8%)	1425 (34.5%)
Obesity (BMI ≥ 30 kg/m^2^)	1311 (15.8%)	600 (14.4%)	711 (17.2%)
Cholesterol, mmol/L	5.6 (4.9, 6.3)	5.6 (4.9, 6.3)	5.5 (4.8, 6.3)
Hypertension	2824 (34.2%)	1652 (39.9%)	1172 (28.4%)
Systolic blood pressure (mm Hg)	126.0 (114.0, 141.0)	131.0 (120.0, 144.0)	119.0 (109.0, 136.0)
Diabetes	304 (3.7%)	172 (4.2%)	132 (3.2%)
Glucose, mmol/L	4.7 (4.3, 5.1)	4.8 (4.5, 5.3)	4.6 (4.2, 5.0)
Myocardial infarction	488 (5.9%)	318 (7.6%)	170 (4.1%)
Stroke	77 (0.9%)	46 (1.1%)	31 (0.7%)
Atrial fibrillation	76 (0.9%)	55 (1.3%)	21 (0.5%)
**Anthropometric measures**			
Body mass index (BMI), kg/m	25.7 (23.2, 28.4)	26.0 (23.8, 28.5)	25.2 (22.6, 28.4)
Waist circumference, cm	88.0 (79.0, 97.0)	93.5 (86.0, 101.0)	82.0 (74.0, 91.0)
Waist-hip ratio	0.88 (0.81, 0.95)	0.94 (0.89, 0.99)	0.82 (0.77, 0.87)
Body shape index, m^11/6^/kg^2/3^ × 1000	76.6 (72.3, 80.7)	79.4 (76.3, 82.4)	73.1 (69.8, 77.0)
Weight-adjusted-waist index, m/kg^2^ × 100	10.0 (9.4, 10.6)	10.2 (9.7, 10.7)	9.7 (9.1, 10.4)
Body roundness index	3.5 (2.6, 4.6)	3.8 (3.0, 4.7)	3.2 (2.3, 4.4)
Relative fat mass	29.4 (25.1, 35.4)	25.7 (22.3, 28.7)	35.1 (30.4, 39.7)

Continuous variables are presented as medians (25th–75th percentile) and categorical variables as n (%).

**Figure 2:**
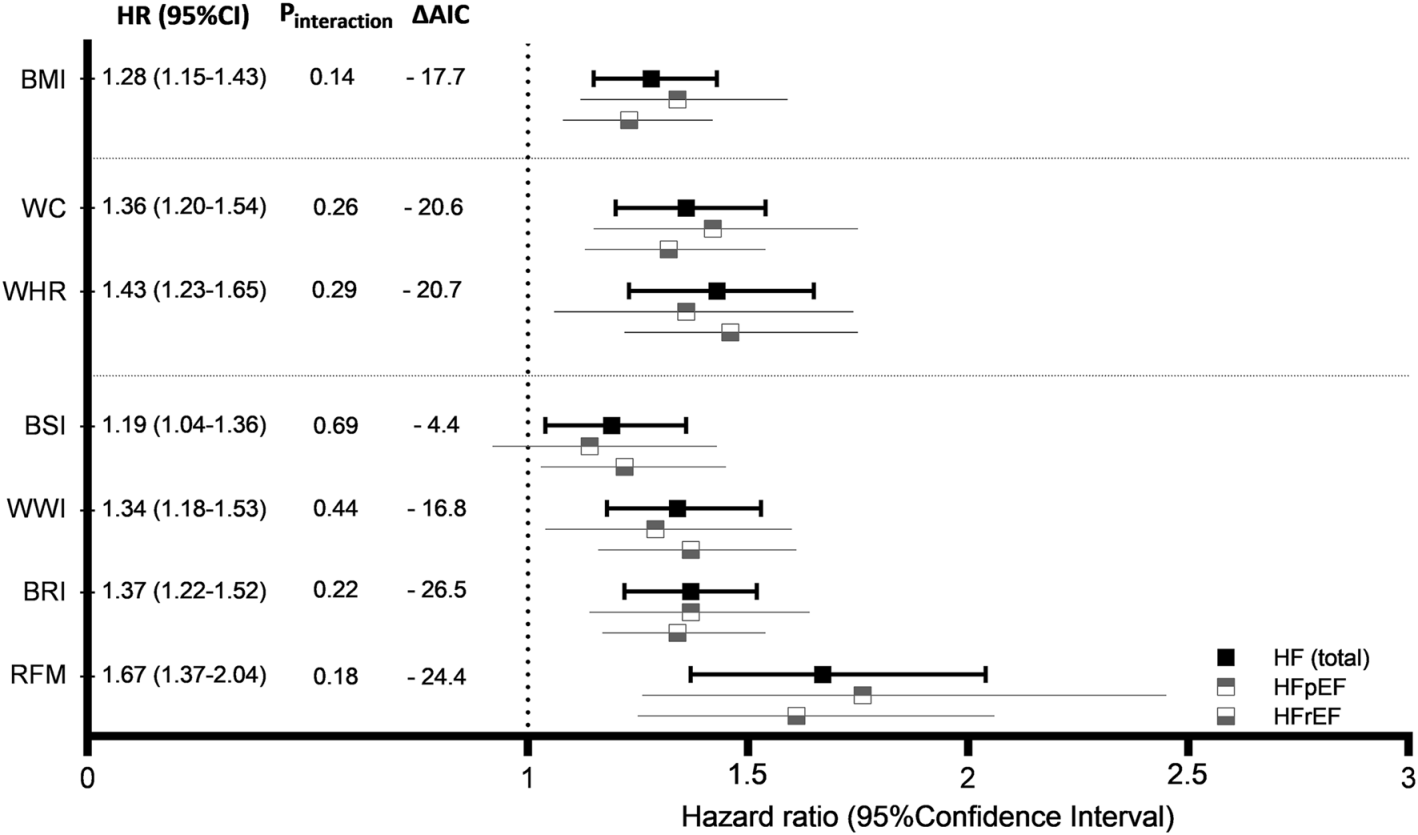
Associations of adiposity indices with incident heart failure. Models were adjusted for age, sex, smoking, cholesterol, systolic blood pressure, glucose, and history of myocardial infarction, stroke and atrial fibrillation. HR represents the hazard ratio per standard deviation change in adiposity index; CI represents confidence interval; P_interaction_ represents the P-value for sex × covariate interaction. *AIC* Akaike information criterion, *BMI* body-mass index, *BRI* body roundness index, *BSI* body shape index, *HF* heart failure, *HFrEF* HF with reduced ejection fraction, *HFpEF* HF with preserved ejection fraction, *RFM* relative fat mass, *WC* waist circumference, *WHR* waist-to-hip ratio, *WWI* weight-adjusted-waist index.

During a mean follow-up of 11.3 ± 3.1 years, 363 individuals (4.1%) developed HF. The overall incidence rate of HF was 3.88 per 1000 person-years (5.13 per 1000 person-years in men and 2.67 per 1000 person-years in women). In multivariable Cox regression models, all adiposity indices, except BSI, were significantly associated with incident HF (P < 0.001) (Table 2, Supplementary Fig. S1). While a unit change in standardized BMI was associated with a 28% increased risk of developing HF [hazard ratio per 1-SD increase (HR) 1.28; 95% confidence interval (CI) 1.15–1.43], an equivalent change in RFM was associated with a 67% increased risk [HR 1.67; 95% CI 1.37–2.04]. Sex did not significantly modify the association of any adiposity index with incident HF (P_int_ > 0.1) (Fig. 2). To check the robustness of results derived from primary analysis, we performed two sensitivity analyses: (i) we substituted glucose and systolic blood pressure with diabetes and hypertension in multivariable models, and (ii) we accounted for death as a competing risk. In both cases, patterns of associations between adiposity indices and incident HF were similar to that observed in the primary analysis (Supplementary Tables S3, S4).

**Table 2:** Associations of adiposity indices with incident heart failure and its subtypes.

	Age-sex adjusted		Multivariable adjusted		Sex-interaction	
	HR (95% CI)	*P*-value	HR (95% CI)	*P*-value	HR_int_ (95% CI)	*P*_int_-value
**Heart failure**						
BMI	1.39 (1.26, 1.54)	< 0.001	1.28 (1.15, 1.43)	< 0.001	0.85 (0.69, 1.05)	0.14
WC	1.49 (1.32, 1.68)	< 0.001	1.36 (1.20, 1.54)	< 0.001	0.87 (0.68, 1.11)	0.26
WHR	1.57 (1.37, 1.80)	< 0.001	1.43 (1.23, 1.65)	< 0.001	0.86 (0.65, 1.14)	0.29
BSI	1.25 (1.10, 1.43)	< 0.001	1.19 (1.04, 1.36)	0.011	0.95 (0.74, 1.22)	0.69
WWI	1.44 (1.27, 1.63)	< 0.001	1.34 (1.18, 1.53)	< 0.001	0.91 (0.72, 1.16)	0.44
BRI	1.46 (1.32, 1.62)	< 0.001	1.37 (1.22, 1.52)	< 0.001	0.88 (0.71, 1.08)	0.22
RFM	1.93 (1.60, 2.33)	< 0.001	1.67 (1.37, 2.04)	< 0.001	0.78 (0.54, 1.12)	0.18
**HFpEF**						
BMI	1.46 (1.24, 1.72)	< 0.001	1.34 (1.12, 1.59)	0.001	0.95 (0.67, 1.37)	0.80
WC	1.56 (1.28, 1.90)	< 0.001	1.42 (1.15, 1.75)	0.001	0.99 (0.66, 1.50)	0.97
WHR	1.48 (1.17, 1.86)	0.001	1.36 (1.06, 1.74)	0.014	1.19 (0.73, 1.94)	0.48
BSI	1.20 (0.97, 1.48)	0.092	1.14 (0.92, 1.43)	0.235	1.04 (0.66, 1.62)	0.88
WWI	1.38 (1.13, 1.69)	0.002	1.29 (1.04, 1.60)	0.020	1.03 (0.67, 1.58)	0.88
BRI	1.48 (1.25, 1.75)	< 0.001	1.37 (1.14, 1.64)	0.001	1.00 (0.70, 1.43)	0.99
RFM	2.04 (1.48, 2.81)	< 0.001	1.76 (1.26, 2.45)	0.001	0.99 (0.51, 1.91)	0.98
**HFrEF**						
BMI	1.34 (1.18, 1.52)	< 0.001	1.23 (1.08, 1.42)	0.003	0.79 (0.60, 1.04)	0.09
WC	1.44 (1.24, 1.67)	< 0.001	1.32 (1.13, 1.54)	0.001	0.79 (0.57, 1.08)	0.13
WHR	1.61 (1.36, 1.91)	< 0.001	1.46 (1.22, 1.75)	< 0.001	0.70 (0.49, 1.01)	0.06
BSI	1.29 (1.10, 1.52)	0.002	1.22 (1.03, 1.45)	0.019	0.90 (0.65, 1.24)	0.51
WWI	1.46 (1.25, 1.71)	< 0.001	1.37 (1.16, 1.61)	< 0.001	0.83 (0.61, 1.12)	0.23
BRI	1.43 (1.26, 1.63)	< 0.001	1.34 (1.17, 1.54)	< 0.001	0.82 (0.63, 1.07)	0.14
RFM	1.84 (1.46, 2.32)	< 0.001	1.61 (1.25, 2.06)	< 0.001	0.65 (0.41, 1.03)	0.07

Multivariable models were adjusted for age, sex, smoking, cholesterol, systolic blood pressure, glucose, and history of myocardial infarction, stroke and atrial fibrillation. HR represents the hazard ratio per standard deviation change in adiposity index; CI represents confidence interval; P_int_ represents the P-value for sex × covariate interaction. A hazard ratio for interaction (HR_int_) > 1 indicates stronger associations in women. A HR_int_ < 1 indicates stronger associations in men.

*BMI* body-mass index, *BRI* body roundness index, *BSI* body shape index, *HF* heart failure, *HFrEF* HF with reduced ejection fraction, *HFpEF* HF with preserved ejection fraction, *RFM* relative fat mass, *WC* waist circumference, *WHR* waist-to-hip ratio, *WWI* weight-adjusted-waist index.

Associations of adiposity indices with incident HF across age categories, and across BMI categories are shown in Tables 3 and 4. When multivariable models were further adjusted for BMI, all adiposity indices except WC remained significantly associated with incident HF (Supplementary Table S5). Largest effect sizes were again observed with RFM [HR 1.59, 95% CI (1.12–2.25)].

**Table 3:** Associations of adiposity indices with incident heart failure across age categories.

	Young (< 55 years) n = 5847		Middle-aged (55–65 years) n = 1459		Old (≥ 65 years) n = 1349	
HF cases	67		108		188	
Incidence rate	1.05		6.48		14.10	

	HR (95% CI)	*P*-value	HR (95% CI)	*P*-value	HR (95% CI)	*P*-value
BMI	1.23 (0.98, 1.55)	0.075	1.30 (1.07, 1.58)	0.007	1.25 (1.05, 1.48)	0.011
WC	1.40 (1.07, 1.84)	0.014	1.27 (1.01–1.60)	0.041	1.34 (1.11–1.62)	0.002
WHR	1.60 (1.17, 2.20)	0.004	1.39 (1.06, 1.82)	0.019	1.32 (1.08, 1.63)	0.007
BSI	1.41 (1.05, 1.90)	0.024	1.00 (0.78, 1.29)	0.999	1.22 (1.01, 1.47)	0.038
WWI	1.52 (1.14, 2.04)	0.005	1.19 (0.93, 1.52)	0.156	1.31 (1.09, 1.58)	0.004
BRI	1.39 (1.09, 1.76)	0.007	1.30 (1.06, 1.58)	0.010	1.34 (1.14, 1.58)	< 0.001
RFM	1.71 (1.15, 2.56)	0.009	1.55 (1.07, 2.23)	0.019	1.58 (1.16, 2.14)	0.003

Incidence rate is presented as HF cases per 1000-person years. Multivariable models were adjusted for age, sex, smoking, cholesterol, systolic blood pressure, glucose, and history of myocardial infarction, stroke and atrial fibrillation. Abbreviations same as in Table 2.

**Table 4:** Associations of adiposity indices with incident heart failure across body-mass index categories.

	Lean (18.5–25 kg/m^2^) n = 3568		Overweight (25–30 kg/m^2^) n = 3416		Obese (BMI ≥ 30 kg/m^2^) n = 1311	
HF cases	71		180		112	
Incidence rate	1.75		4.67		7.69	

	HR (95% CI)	*P*-value	HR (95% CI)	*P*-value	HR (95% CI)	*P*-value
BMI	0.94 (0.47, 1.88)	0.859	1.14 (0.73, 1.77)	0.569	1.13 (0.87, 1.48)	0.356
WC	1.03 (0.66, 1.63)	0.883	1.30 (0.98, 1.73)	0.074	1.21 (0.91, 1.61)	0.185
WHR	1.07 (0.74, 1.55)	0.721	1.46 (1.16, 1.85)	0.001	1.26 (0.95, 1.67)	0.105
BSI	1.08 (0.81, 1.43)	0.609	1.27 (1.04, 1.55)	0.018	1.16 (0.89, 1.51)	0.267
WWI	1.10 (0.81, 1.50)	0.533	1.37 (1.11, 1.68)	0.003	1.21 (0.93, 1.57)	0.162
BRI	1.05 (0.63, 1.74)	0.859	1.50 (1.14, 1.97)	0.004	1.21 (0.96, 1.53)	0.105
RFM	1.25 (0.73, 2.13)	0.418	1.80 (1.14, 2.85)	0.012	1.55 (0.86, 2.79)	0.142

Multivariable models were adjusted for age, sex, smoking, cholesterol, systolic blood pressure, glucose, and history of myocardial infarction, stroke and atrial fibrillation. Abbreviations same as in Table 2.

Among incident HF cases, a total of 120 individuals were classified as HFpEF and 243 were classified as HFrEF. The overall incidence rate of HFpEF was 1.27 per 1000 person-years (1.20 per 1000 person-years in men and 1.34 per 1000 person-years in women), and that of HFrEF was 2.58 per 1000 person-years (3.90 per 1000 person-years in men and 1.32 per 1000 person-years in women). The majority of adiposity indices were strongly associated with both HFpEF and HFrEF in the total population. Amongst adiposity indices, RFM showed strongest associations with both HFpEF [HR 1.76, 95% CI (1.26–2.45)] and HFrEF [HR 1.61, 95% CI (1.25–2.06)]. All adiposity indices were similarly associated with incident HFpEF in both sexes, but RFM, WHR and BMI displayed stronger associations with HFrEF in men than in women (P_int_: 0.07, 0.06 and 0.09 respectively) (Table 2).

None of the adiposity indices improved discrimination of HF beyond the clinical model. However, addition of several adiposity indices, including BMI, substantially improved model fit by reducing the prediction error (Table 5, Fig. 2). Strongest improvements were observed after adding BRI and RFM (ΔAIC − 26.5 and − 24.4 respectively). Within HF subtypes, strongest improvement in model fit was observed after adding RFM and BRI for HFpEF, and after adding WHR, BRI and RFM for HFrEF (Table 5).

**Table 5:** Adiposity indices and improvement in model fit.

	C-statistic	ΔC-statistic	AIC	ΔAIC	*P*-value
**Heart failure**					
Base model	0.848	–	5800.5	–	
BMI	0.850	0.002	5782.8	− 17.7	< 0.001
WC	0.851	0.003	5779.9	− 20.6	< 0.001
WHR	0.852	0.004	5779.8	− 20.7	< 0.001
BSI	0.850	0.002	5796.1	− 4.4	0.041
WWI	0.852	0.004	5783.7	− 16.8	< 0.001
BRI	0.852	0.004	5774.0	− 26.5	< 0.001
RFM	0.851	0.003	5776.1	− 24.4	< 0.001
**HFpEF**					
Base model	0.853	–	1919.4	–	
BMI	0.855	0.002	1911.4	− 8.0	0.007
WC	0.856	0.003	1911.4	− 8.0	0.007
WHR	0.856	0.003	1915.6	− 3.8	0.053
BSI	0.855	0.002	1920.0	+ 0.6	0.498
WWI	0.856	0.003	1916.2	− 3.2	0.072
BRI	0.857	0.004	1910.8	− 8.6	0.005
RFM	0.856	0.003	1910.0	− 9.4	0.003
**HFrEF**					
Base model	0.848	–	3873.0	–	
BMI	0.850	0.002	3866.4	− 6.6	0.014
WC	0.851	0.003	3863.3	− 9.7	0.003
WHR	0.852	0.004	3858.4	− 14.6	< 0.001
BSI	0.850	0.002	3869.6	− 3.4	0.066
WWI	0.852	0.004	3861.6	− 11.4	0.001
BRI	0.852	0.004	3859.1	− 13.9	< 0.001
RFM	0.851	0.003	3860.6	− 12.4	< 0.001

AIC represents Akaike information criterion. All other abbreviations are same as in Table 2. P-values are based on likelihood ratio test. If the reduction in AIC is less than 2: no substantial evidence to support the candidate model; between 4 and 7: candidate model has considerably less support; greater than 10: no support for the candidate model.

## Discussion

In the current study including 8295 community-dwelling adults, most of the novel as well as established indices of adiposity were strongly associated with incident HF. Amongst adiposity indices, we found that RFM displayed the strongest associations with incident HF.

RFM is a newly-developed parameter of adiposity that more accurately estimates whole-body fat percentage compared to traditional equations based on BMI or WHR^16^. From a practical standpoint, it is interesting to observe that fat percentage is higher in women than in men^27,28^, and average RFM values are also higher in women than in men; for instance, in the PREVEND cohort, mean RFM was around 35% in women and around 25% in men. By contrast, most existing measures of adiposity are, on average, higher in men than in women (Table 1). With regard to wide-spread applicability, RFM could potentially be used in routine clinical practice or public health surveillance programmes—even in resource poor settings. This is because RFM not only correlates strongly with HF risk, but can also be calculated using a relatively simple formula, requiring only height and waist circumference—both of which could be determined using a measuring tape.

In subgroup analyses, adiposity indices tended to be more strongly associated with incident HF in younger individuals (i.e., < 55 years). RFM, however, displayed the strongest relative risk across all age groups, including older individuals (i.e., ≥ 65 years). Across BMI categories, adiposity indices, in general, were more strongly associated with incident HF in overweight individuals. Again, RFM had the strongest relative risk across all BMI categories, including in those classified as obese. Most indices of adiposity were also associated with individual HF subtypes, and RFM consistently displayed the largest effect sizes for both HFrEF and HFpEF.

Sex-related differences in associations of BMI with incident HF has been previously reported; while some studies showed stronger associations in men than in women^22^, other studies showed neutral^9^ or even opposite trends^8^. In the current study, we report that both novel as well as established indices of adiposity were similarly associated with incident HF in both sexes. Among HF subtypes, we found that most measures of adiposity were strongly and similarly associated with incident HFpEF in both sexes, suggesting that excessive fat accumulation predisposes both men and women to HFpEF. On the other hand, RFM, BMI and WHR tended to be more strongly associated with HFrEF development in men. Similar trends have been observed for BMI and WC in a meta-analysis of four large cohorts including the PREVEND study^29^, highlighting that adiposity may be a stronger risk factor for HFrEF in men than in women.

Interestingly, we found that none of the adiposity indices substantially improved discrimination of the clinical HF model. One potential explanation could be that the C-statistic is a less sensitive tool to measure improvement in model fit, especially if the base model already has excellent discrimination^22,30^. Using more global and sensitive measures of model fit such as LHR χ^2^ statistic and AIC, we show that RFM and BRI may be more useful than BMI in improving HF risk prediction. Given that RFM and BRI also consistently improved prediction of both HFrEF and HFpEF, future studies should evaluate the potential of including these indices in HF risk estimation.

In summary, we report for the first time, associations between newly-developed adiposity indices (i.e., RFM, BRI and WWI) and incident HF in a large well-characterized cohort. A particular strength of our study was that the HF endpoint was adjudicated, and LVEF was available for all HF cases. The long term follow-up of participants and a 1:1 sex ratio further strengthen our analyses. Nevertheless, we acknowledge that PREVEND is a relatively young cohort with low overall event rates. Another limitation is that the PREVEND study, by design, included a higher proportion of individuals with UAE > 10 mg/L. Finally, the current study was conducted on a predominantly White population from the northern Netherlands, warranting validation of our findings in cohorts from other geographical locations and ethnicities.

## Conclusion

Amongst indices of adiposity, relative fat mass displayed the strongest association with HF risk in community-dwelling adults. Future studies should examine the value of including relative fat mass in HF risk prediction models.

## Supplementary Information

Supplementary Information.
