# Supplementary material for: Relative fat mass, a new index of adiposity, is strongly associated with incident heart failure: data from PREVEND
Source: Sci Rep. 2022 Jan 7;12:147. doi: 10.1038/s41598-021-02409-6 (PMC8741934; doi:10.1038/s41598-021-02409-6)
Supplement: Supplementary file 1 — Supplementary Information. [file 41598_2021_2409_MOESM1_ESM.pdf]

Relative Fat Mass, a Newly Developed Index of Adiposity, is Strongly Associated with Incident Heart Failure: Data from the PREVEND Study

Navin Suthahar* MD MSc PhD[1], Laura M.G. Meems MD PhD[1], Coenraad Withaar MSc[1], Thomas M. Gorter MD PhD[1], Lyanne M. Kieneker PhD[2], Ron T. Gansevoort MD PhD[2], Stephan J.L. Bakker MD PhD[2], Dirk J. van Veldhuisen MD PhD[1], Rudolf A. de Boer* MD PhD[1]

Affiliation:

[1] University of Groningen, University Medical Center Groningen, Department of Cardiology, the Netherlands.

[2] University of Groningen, University Medical Center Groningen, Division of Nephrology, Department of Internal Medicine, the Netherlands.

To whom correspondence should be addressed:

Navin Suthahar MD MSc PhD: n.suthahar@umcg.nl

Rudolf A. de Boer MD PhD : r.a.de.boer@umcg.nl

University Medical Centre Groningen

Department of Cardiology, AB 31

Hanzeplein 1, 9713GZ, Groningen, the Netherlands

Phone: +31503612355 / Fax: +31503615525

Supplementary Table 1. Distribution of adiposity indices in the PREVEND cohort

**MEN**	**P (10)**	**P (25)**	**P (50)**	**P (75)**	**P (90)**	**Mean (SD)**
BMI ($kg.m^{-2}$)	22.0	23.8	26.0	28.5	30.9	26.3 (3.6)
WC (m)*100	80.0	86.0	93.5	101.0	108.0	94.0 (11.0)
WHR	0.85	0.89	0.94	0.99	1.03	0.94 (0.07)
BSI ($m^{11/6}.kg^{-2/3}$)*1000	73.6	76.3	79.4	82.4	85.3	79.4 (4.7)
WWI ($m.kg^{-2}$)*100	9.3	9.7	10.2	10.7	11.2	10.2 (0.74)
BRI	2.4	3.0	3.8	4.7	5.6	3.9 (1.3)
RFM	18.9	22.3	25.7	28.7	31.1	25.3 (4.7)
**WOMEN**	**P (10)**	**P (25)**	**P (50)**	**P (75)**	**P (90)**	**Mean (SD)**
BMI ($kg.m^{-2}$)	21.0	22.6	25.2	28.4	32.2	26.0 (4.6)
WC (m)*100	69.0	74.0	82.0	91.0	100.5	83.4 (12.5)
WHR	0.73	0.77	0.82	0.87	0.93	0.82 (0.08)
BSI ($m^{11/6}.kg^{-2/3}$)*1000	67.2	69.8	73.1	77.0	81.2	73.7 (5.7)
WWI ($m.kg^{-2}$)*100	8.7	9.1	9.7	10.4	11.1	9.8 (0.9)
BRI	1.8	2.3	3.2	4.4	5.8	3.5 (1.6)
RFM	27.0	30.4	35.1	39.7	43.5	35.1 (6.2)

Abbreviations: BMI, body-mass index; BRI, body roundness index; BSI, body shape index; RFM, relative fat mass; WC, waist circumference; WHR, waist-to-hip ratio; WWI, weight-adjusted-waist index.

Supplementary Table 2. Correlation of adiposity indices with each other and with age

**MEN**	BMI	WC	WHR	BSI	WWI	BRI	RFM	Age
BMI	1.00							
WC	0.86	1.00						
WHR	0.62	0.83	1.00					
BSI	0.22	0.66	0.77	1.00				
WWI	0.53	0.79	0.87	0.90	1.00			
BRI	0.87	0.94	0.85	0.63	0.87	1.00		
RFM	0.84	0.93	0.86	0.67	0.89	0.97	1.00	
Age	0.21	0.37	0.44	0.52	0.58	0.45	0.47	1.00
**WOMEN**	BMI	WC	WHR	BSI	WWI	BRI	RFM	Age
BMI	1.00							
WC	0.85	1.00						
WHR	0.44	0.76	1.00					
BSI	0.21	0.67	0.84	1.00				
WWI	0.52	0.84	0.87	0.91	1.00			
BRI	0.86	0.96	0.74	0.64	0.87	1.00		
RFM	0.84	0.95	0.77	0.67	0.88	0.96	1.00	
Age	0.31	0.38	0.34	0.36	0.47	0.43	0.47	1.00

Abbreviations same as in Table S1.

Supplementary Table 3. Associations of adiposity indices with incident heart failure (Sensitivity Analysis 1)

	Multivariable adjusted		Sex-interaction	
	**HR (95% CI)**	***P*-value**	**HR_{int} (95% CI)**	**P_{int}-value**
BMI	1.26 (1.13, 1.40)	<0.001	0.87 (0.71, 1.07)	0.194
WC	1.32 (1.17, 1.50)	<0.001	0.87 (0.69, 1.11)	0.274
WHR	1.39 (1.21, 1.61)	<0.001	0.85 (0.64, 1.13)	0.268
BSI	1.18 (1.03, 1.35)	0.015	0.93 (0.72, 1.21)	0.596
WWI	1.33 (1.17, 1.52)	<0.001	0.90 (0.70, 1.14)	0.382
BRI	1.34 (1.20, 1.49)	<0.001	0.88 (0.72, 1.09)	0.246
RFM	1.62 (1.33, 1.97)	<0.001	0.79 (0.55, 1.14)	0.215

Multivariable models were adjusted for age, sex, smoking, cholesterol, **hypertension, diabetes,** and history of myocardial infarction, stroke and atrial fibrillation. HR represents the hazard ratio per standard deviation change in adiposity index; CI represents confidence interval; P_{int} represents the P-value for sex*covariate interaction. A hazard ratio for interaction (HR_{int}) > 1 indicates stronger associations in women. A HR_{int} < 1 indicates stronger associations in men. Other abbreviations same as in Supplementary Table 1.

Supplementary Table 4. Associations of adiposity indices with incident heart failure after accounting for death as a competing risk (Sensitivity Analysis 2)

	Multivariable adjusted		Sex-interaction	
	**HR (95% CI)**	***P*-value**	**HR_{int} (95% CI)**	**P_{int}-value**
BMI	1.30 (1.17, 1.45)	<0.001	0.87 (0.71, 1.07)	0.194
WC	1.36 (1.20, 1.54)	<0.001	0.91 (0.72, 1.16)	0.274
WHR	1.41 (1.22, 1.63)	<0.001	0.92 (0.70, 1.21)	0.268
BSI	1.16 (1.02, 1.33)	0.026	0.99 (0.78, 1.26)	0.596
WWI	1.32 (1.16, 1.51)	<0.001	0.94 (0.74, 1.19)	0.382
BRI	1.36 (1.22, 1.52)	<0.001	0.91 (0.74, 1.12)	0.246
RFM	1.69 (1.38, 2.06)	<0.001	0.82 (0.56, 1.18)	0.215

Multivariable models accounted for death as a competing risk, and were adjusted for age, sex, smoking, cholesterol, systolic blood pressure, glucose, and history of myocardial infarction, stroke and atrial fibrillation. HR represents the hazard ratio per standard deviation change in adiposity index; CI represents confidence interval; P_{int} represents the P-value for sex*covariate interaction. A hazard ratio for interaction (HR_{int}) > 1 indicates stronger associations in women. A HR_{int} < 1 indicates stronger associations in men. Other abbreviations same as in Supplementary Table 1.

Supplementary Table 5. Associations of adiposity indices with incident heart failure after adjusting for body-mass index

	**HR (95% CI)**	**P-value**
WC	1.24 (1.00, 1.55)	0.051
WHR	1.31 (1.11, 1.53)	0.001
BSI	1.19 (1.04, 1.36)	0.014
WWI	1.23 (1.07, 1.43)	0.004
BRI	1.39 (1.12, 1.71)	0.003
RFM	1.59 (1.12, 2.25)	0.009

Abbreviations same as in Table S1.

Supplementary Figure 1. Multivariable fractional polynomial models depicting associations of novel adiposity indices with incident heart failure

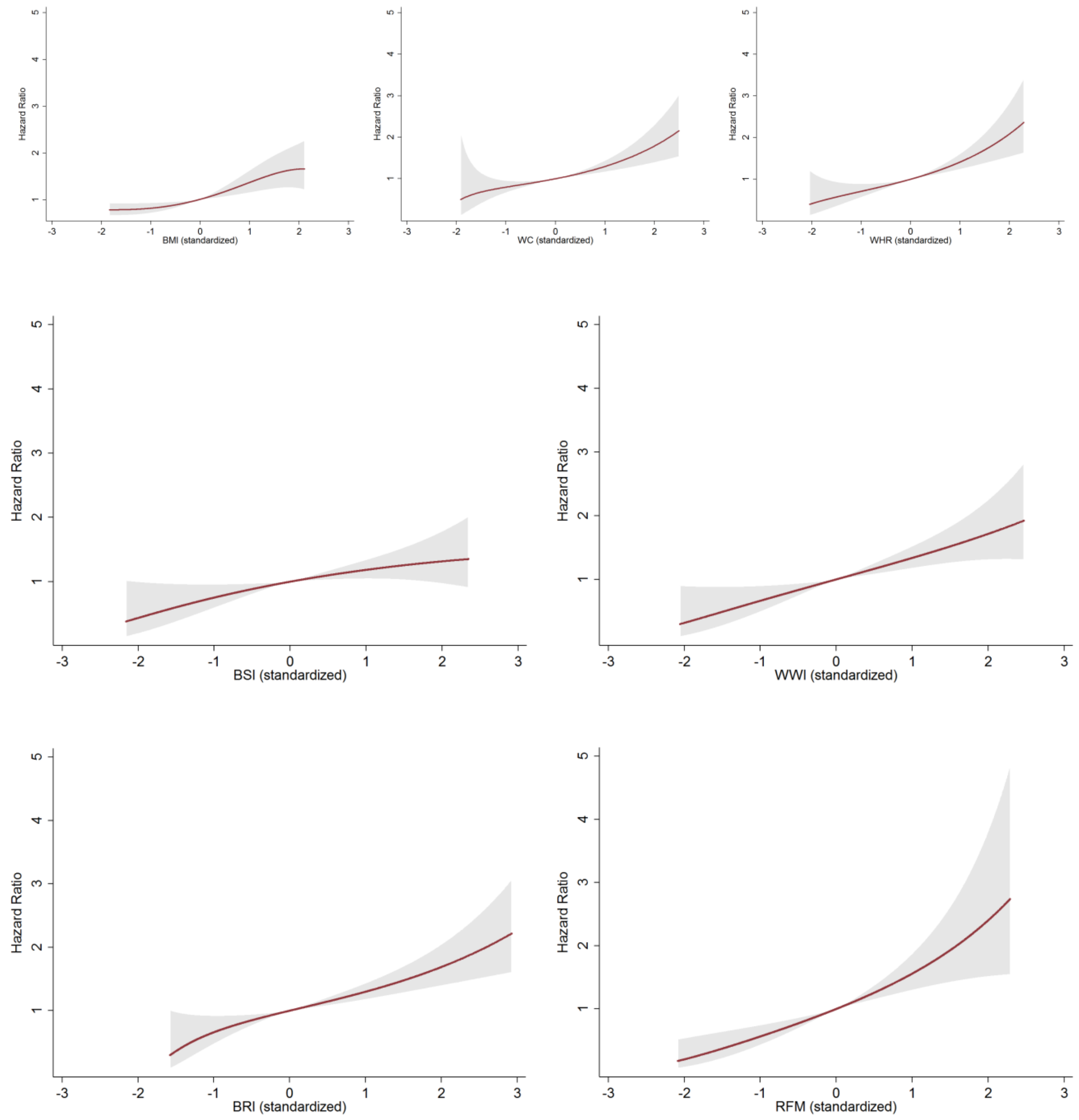

Fractional polynomial models are adjusted for age, sex, smoking, cholesterol, systolic blood pressure, glucose, and history of myocardial infarction, stroke and atrial fibrillation. Abbreviations: BMI, body-mass index; BRI, body roundness index; BSI, body shape index; RFM, relative fat mass; WC, waist circumference; WHR, waist-to-hip ratio; WWI, weight-adjusted-waist index.
